# Real-World Effectiveness of COVID-19 Vaccines (ChAdOx-1s, CoronaVac, BBIBP-CorV, mRNA-1273, and BNT162b2) in Jakarta: Protocol for Test-Negative Design of Health Care Data

**DOI:** 10.2196/56519

**Published:** 2025-04-10

**Authors:** Erlina Burhan, Farchan Azzumar, Fira Alyssa Gabriella Sinuraya, Muhammad Ilham Dhiya Rakasiwi, Ihya Akbar, Farhan Mubarak, Anggit Tresna Rengganis, Rizky Abi Rachmadi, Hera Afidjati

**Affiliations:** 1 Department of Pulmonology and Respiratory Medicine Faculty of Medicine Universitas Indonesia-Persahabatan Hospital Jakarta Indonesia; 2 Indonesian Society of Respirology Jakarta Indonesia; 3 Respiratory Programmatic Implementation and Research Institute Jakarta Indonesia; 4 Faculty of Public Health Universitas Indonesia Depok Indonesia

**Keywords:** COVID-19, protocol, vaccine, vaccine effectiveness, Delta, Omicron

## Abstract

**Background:**

ChAdOx-1s, CoronaVac, BBIBP-CorV, mRNA-1273, and BNT162b2 are the five common COVID-19 vaccines used in Jakarta. Randomized controlled trials have provided robust evidence of the safety and efficacy profile of these vaccines, but their real-world vaccine effectiveness against symptomatic COVID-19 and deaths in communities with social inequalities and health care constraints remains unclear.

**Objective:**

This study aims to evaluate the real-world effectiveness of these COVID-19 vaccines during the waves associated with the Delta and Omicron variants by analyzing existing electronic health care sources.

**Methods:**

A population-based study with a test-negative case-control design will be used to evaluate COVID-19 vaccine effectiveness in Jakarta, focusing on the Delta and Omicron waves. It includes adults 18 years and older who underwent reverse transcription polymerase chain reaction testing for symptomatic COVID-19, classifying them as cases or controls based on their test results. The analysis will consider multiple COVID-19 vaccines introduced during these periods, with participants categorized by vaccination status. Several potential confounders will be assessed, including demographic factors and comorbidities. Data will be linked from various health datasets, and statistical analyses will be performed to determine vaccine effectiveness and potential waning immunity over time. After data linkage, patients’ identities will be encrypted.

**Results:**

The research, funded from 2022 to 2024, involved proposal preparation and ethical review in 2023 and enrollment from early 2024 to July 2024, resulting in about 4 million linked data points. Data analysis is ongoing, with initial results expected for publication in early 2025.

**Conclusions:**

This study will be the first to evaluate the effectiveness of different types of COVID-19 vaccines (inactivated, viral-vector, and mRNA) used in Jakarta during the pandemic, providing valuable scientific evidence to inform future vaccination strategies in the country.

**International Registered Report Identifier (IRRID):**

DERR1-10.2196/56519

## Introduction

### Background

Since its emergence in December 2019, SARS-CoV-2, which is the virus that causes COVID-19, has been continuously spreading worldwide [1]. As of November 12, 2021, this pandemic has caused more than 250 million cases and more than 5 million deaths over the previous 2 years [2]. Vaccination programs are one of the most successful public health interventions that primarily aim to develop herd immunity and protection against pathogens. To date, the World Health Organization has approved 8 COVID-19 vaccines that are efficacious in preventing infection, reducing the level of severity and the number of deaths due to SARS-CoV-2 infection [3]. However, most data were generated from clinical trial studies that were different from a real-world setting due to their well-controlled nature [4]. Furthermore, real-world vaccine effectiveness (VE) studies were mostly conducted in high-income nations, and few studies came from low- and middle-income countries, even though these countries might have unique social and economic characteristics that not only affect vaccination acceptance but also disease outcomes [5]. A previous study has reported the association of living in an impoverished neighborhood with an increased risk for mortality even after COVID-19 vaccination [6]. Therefore, studies investigating how these COVID-19 vaccines might perform in real-world settings where social inequalities exist are still essential for informing policies and strategies to improve disease outcomes in future pandemics, especially in societies with health care system constraints [7]. This study aims to evaluate COVID-19 VE against symptomatic and fatal SARS-CoV-2 infection during the Delta and Omicron waves in Jakarta, where income inequality and socioeconomic segregation have become a common phenomenon [8].

### Study Objectives

The primary objective is to study real-world VE against symptomatic COVID-19 (mild, moderate, and severe).

The following secondary objectives were also explored:

- Real-world VE against mortality 30 days after the first positive COVID-19 result
- Real-world VE based on vaccination status at the time of the reverse transcription polymerase chain reaction (RT-PCR) test
- Real-world VE based on the time since receipt of the last vaccine dosage

## Methods

### Study Design and Setting

This population-based study adopts a test-negative case-control design to analyze COVID-19 VE against symptomatic and fatal SARS-CoV-2 infection from available health care datasets in the Special Capital Region of Jakarta, one of the provinces with the highest population density (15,900 people per km^2^ in 2020) and case rate per population relative to other regions in Indonesia [9]. Despite its robust development and economic growth, the contrast between rich and poor neighborhoods was highly prevalent in many parts of Jakarta’s districts [8,10].

**Figure 1:**
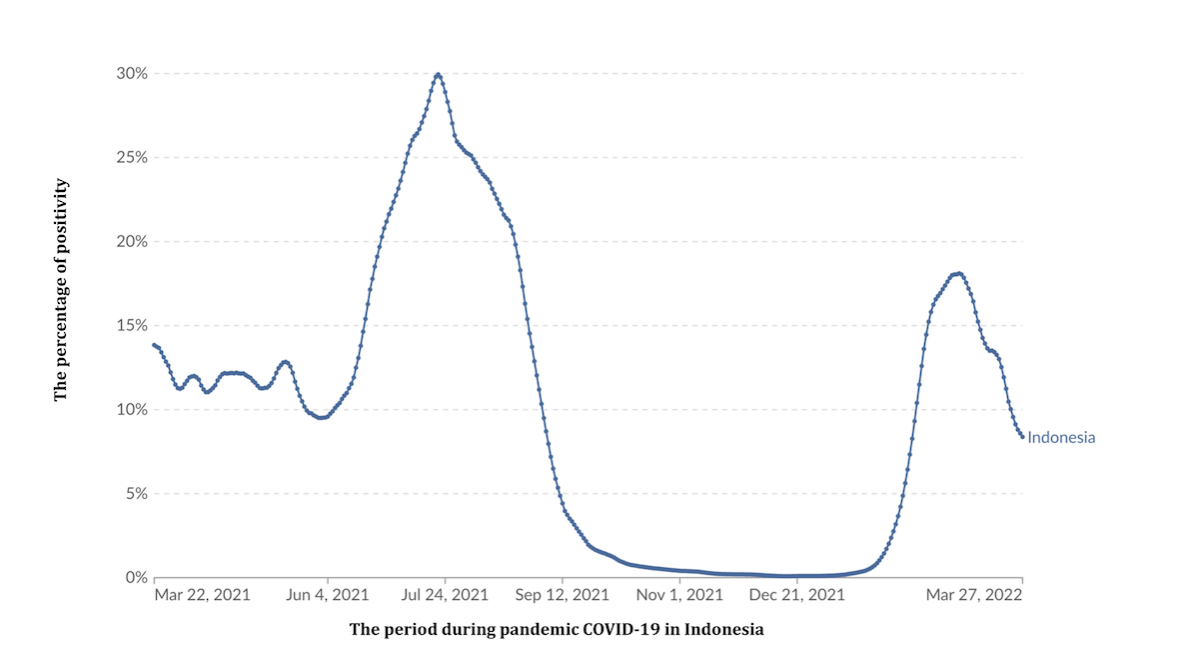
Trend of the national COVID-19 positivity rate in Indonesia from 2021 to 2022. This modified image is cited from Our World in Data.

This study will estimate VE against COVID-19 during the period of interest, which were the COVID-19 waves associated with the Delta and Omicron variants, from June 1 to August 31, 2021, and January 1 to April 2, 2022, respectively. Within each of these periods, a cycle of sustained upward and downward trends of the test positivity rate was observed [11-15] (Figure 1). Furthermore, the Delta and Omicron variants were estimated to be responsible for around 90% of SARS-CoV-2 infections in Jakarta during these two periods [14,16].

During the Delta and Omicron periods, several brands of COVID-19 vaccines were introduced in Jakarta for vaccine rollout, such as AstraZeneca, SinoVac-CoronaVac Biofarma, Covovax-Novovax, Indovax, Johnson & Johnson, Moderna, Sinopharm BBIBP, and Pfizer (Table S1 in Multimedia Appendix 1) [17]. Among these COVID-19 vaccines, Coronavac-Sinovac was the earliest COVID-19 vaccine to be introduced in January 2021 and used for mass vaccinations of doses 1 and 2, followed by AstraZeneca, Sinopharm, Pfizer, and Moderna in August 2021 [18,19]. Booster vaccinations 6 months after the 2-dose primary vaccination with Moderna and Pfizer vaccines were initially introduced to health care workers in July 2021, followed by the approval of the AstraZeneca vaccine as a booster in the national vaccination program in January 2022 [20,21]. To expedite the booster vaccination coverage, the timeline for booster administration was shortened to 3 months after the 2-dose primary vaccination, and additional inactivated COVID-19 vaccines, such as SinoVac and Sinopharm, were included in the national booster program [22]. Different from Pfizer, Moderna, and AstraZeneca, these inactivated COVID-19 vaccines were only approved as boosters for patients who received similar vaccine platform for their primary vaccination regimen [23]. Until the end of the Omicron period, all of the distributed COVID-19 vaccines in Jakarta were developed from the ancestral strain of SARS-CoV-2.

### Study Participants

The study will include people 18 years or older who domiciled in Jakarta and underwent an RT-PCR test for SARS-CoV-2 infection due to symptoms of influenza-like illnesses within 10 days of the test date. Although people may have more than one RT-PCR result during the period of interest, this study will only account for the first positive or negative result per person as the index test date for each of the COVID-19 waves. People who had at least one sample with a positive SARS-CoV-2 RT-PCR result during the period of interest will be classified as cases, while people with a negative SARS-CoV-2 RT-PCR without any positive results over the period of interest will be classified as controls.

People with a positive RT-PCR test result within 90 days of the preceding index test date, with inconclusive RT-PCR results, who received different vaccines for dose 1 and dose 2 or a heterologous primary vaccine series, or who had a time interval between dose 1 and dose 2 that was less than the government recommendation (<21 days for Pfizer and Sinopharm, <28 days for CoronaVac and Moderna, and <12 weeks AstraZeneca) will be excluded from the analysis. We will also exclude people with incomplete vaccination records, such as people who declared receiving 2 vaccine doses without being able to verify the previous dose.

### Study Variables

The primary outcome in this study is symptomatic SARS-CoV-2 infection, defined as a positive RT-PCR test within 10 days from the onset of influenza-like illness. The secondary outcome in this study is fatal SARS-CoV-2 infection, defined as death within 30 days after a positive RT-PCR test result [24]. Meanwhile, the primary exposure in this study was vaccination status, which can be classified as unvaccinated, dose 1 or partial vaccination, dose 2 or primary vaccination, and booster dose. People without any vaccination entries after data linkage with the PCARE Vaksin dataset at the end of each period of interest will be classified as unvaccinated persons. For vaccinated people, we will only consider the last vaccination data entry that happened before the patients’ index RT-PCR test date.

Several covariates should be assessed as potential confounders in the study, such as age in years at the index test date, gender, calendar week of the RT-PCR collection during the period of interest, living in impoverished neighborhoods, presence of any comorbidities, occupation, and reinfection status. The calendar week of the RT-PCR collection during the period of interest will be presented as a whole number starting from week 1 as the start of the period of interest. Living in an impoverished neighborhood will be presented as a dichotomous variable. People will be classified as living in an impoverished neighborhood if their neighborhood number at the subdistrict level is listed as one of the impoverished neighborhoods in the 2018 Jakarta Governor’s regulation concerning improving the quality of settlements in residential areas. Occupation will be presented as categorical variables that consist of health care worker, public or government official, and civilian. Reinfection or people with previous SARS-CoV-2 infections will be presented as a dichotomous variable. People with a previous positive RT-PCR SARS-CoV-2 result that occurred more than 90 days after the current case will be classified as people with a previous SARS-CoV-2 infection [25].

To control for unmeasured confounders [26], such as societal preventive measures or the changing dynamic of the viral transmission within the community, this study will match each case with a control using a ratio of 1:2 by matching for age within 10 years of the case’s age, gender, and the calendar week of the RT-PCR collection during the period of interest. The matching procedure will be assessed with standardized mean differences, with values less than 0.1 indicating sufficient matching [27,28].

### Data Sources

**Figure 2:**
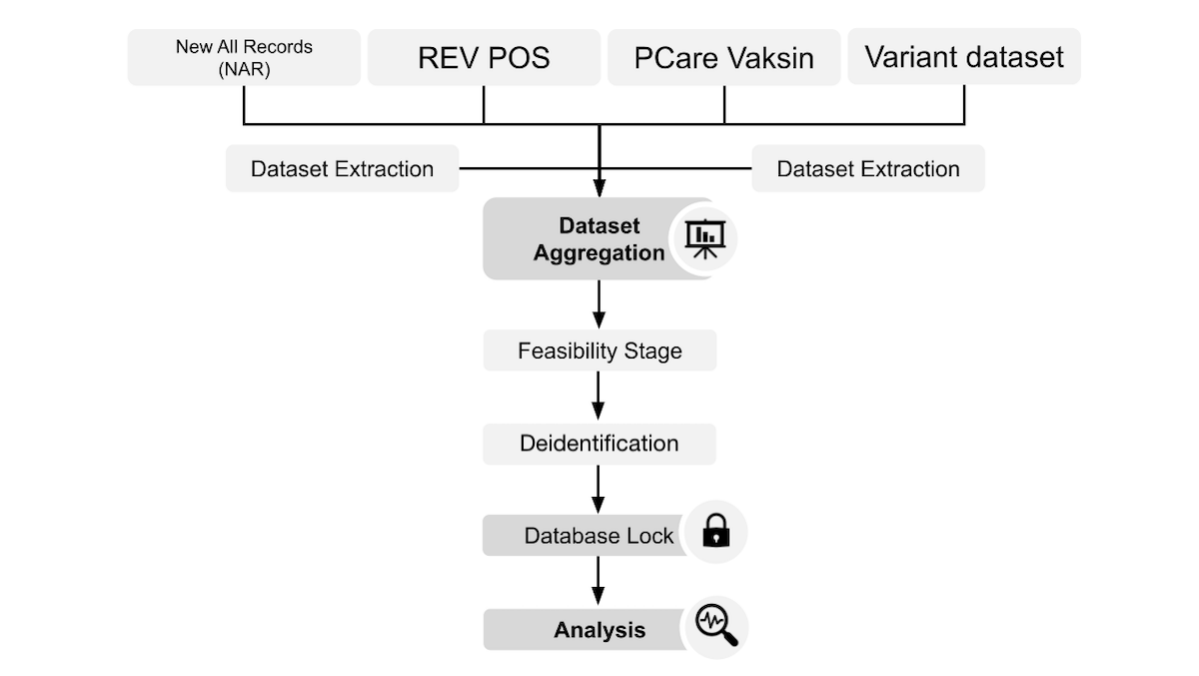
Study workflow. PCare: Primary Care.

This study will analyze the final datasets derived from the integration of several datasets provided by the Jakarta Provincial Health Office, Ministry of Health, and Social Security Agency on Health after April 2022 (Table 1). Variables such as ID number and date of birth will be used for the dataset linkage (Figure 2). From the New All Records (NAR) and Suspect and Probable (SUSPROB) datasets, symptomatic people with a positive or negative first RT-PCR test result within each of the periods of interest will be identified as cases or controls. Furthermore, people identified as cases will be linked to the entries in the REV POS dataset to extract the outcome of their disease course (Figure 2). People with incomplete data for ID number, date of birth, gender, swab collection date, PCR test result, domicile address, outcome, and date of death (if the case ends in death) will be excluded from further data linkage.

**Table 1:** List source for merged datasets.

Name	Developer	Description	Variable identified
NAR^a^	Ministry of Health Republic of Indonesia	Record of all RT-PCR^b^ SARS-CoV-2 tests carried out by affiliated laboratories	Name ID number Date of birth Sex (male, female) Comorbidity Domicile address Laboratory name Reason for testing Swab collection date RT-PCR result confirmation date RT-PCR testing result
SUSPROB^c^	Jakarta Provincial Health Office	Record and follow-ups of all people with influenza-like illness or probable COVID-19	Name ID number Date of birth Sex (male, female) Comorbidity Domicile address Presence of influenza-like illness (fever, chill, cough, etc) Date of symptom onset Travel history Plan swab testing date Swab collection date RT-PCR testing result
REV POS	Jakarta Provincial Health Office	Records and follow-ups on all positive cases in Jakarta	Name ID number Date of birth Sex (male, female) Comorbidity Domicile address Date of swab collection Case severity (asymptomatic, mild, moderate, severe) Outcome (survived or death) Date of death
PCARE^d^ Vaksin	Social Security Agency on Health Indonesia (BPJS Kesehatan)	Record of all administered COVID-19 vaccines and their recipients	Name ID number Date of birth Sex (male, female) Domicile address Occupation or vaccination batch group (health workers, government officials, elderly, children, civilians) Name of health care facility/vaccination center Vaccination date Vaccination ticket number Vaccination dose Vaccine name Vaccine batch/lot number
Variant dataset	Jakarta Provincial Health Office	Sequencing and genotyping results of the randomly picked positive SARS-CoV-2 samples in the Jakarta area	Sample registry number Date of sample collection Name of the sender laboratory Date of the sample arrival at sequencing lab Name of the sequencing lab Date of the sequencing test Date of result confirmation Variant of SARS-CoV-2

^a^NAR: New All Records.

^b^RT-PCR: reverse transcription polymerase chain reaction.

^c^SUSPROB: Suspect and Probable.

^d^PCARE: Primary Care.

People with data linked to the entries within the Primary Care (PCARE) Vaksin dataset will be classified as vaccinated people (Figure 2). To avoid misclassification of foreigners as unvaccinated people, the study will only use entries with national identity card numbers as ID numbers from each of the study datasets. Vaccinated people with incomplete data on vaccination date and vaccine name will be excluded from the final dataset (Table S2 in Multimedia Appendix 1). From the final dataset, a subset of data containing the matched cases and controls will be created (Table S3 in Multimedia Appendix 1).

As genomic testing was not routinely performed in Indonesia, no data linkage will be performed on the variant dataset. In contrast, the variant dataset, which consisted of the genomic test result of the randomly sampled specimen from the population, will be used to confirm the dominant SARS-CoV-2 variant in Jakarta during each period of interest.

### Sample Size

The calculation formula for the sample size used in this study will follow the methodology described by O’Neill [29]. Assuming vaccination coverage between 20%-90%, a precision of ±5%, a type 1 error rate of 0.05, and an anticipated VE of 50%-90%, the minimum sample size needed for each specific COVID-19 vaccine brand and dose VE analysis is roughly around 15,000, with 5000 cases and 10,000 controls [24,29] (Table S4 in Multimedia Appendix 1).

We expect to analyze 400,000-900,000 people after the aggregation of all datasets. Therefore, we should have enough data to conduct the VE analyses for each specific COVID-19 brand and regimen in our final dataset. We will compare the proportion of COVID-19 vaccines received by eligible people in our dataset during each period of interest to those reported by the Ministry of Health [17] (Table S1 in Multimedia Appendix 1). Furthermore, we will exclude people who received COVID-19 vaccine brands with vaccination coverage of less than 20%, as these vaccines are usually distributed briefly in the population or only available to specific subgroups within the community. Moreover, we might consider combining different COVID-19 vaccine brands with the same platform into one group to decrease the required sample size, especially for the subgroup VE analysis [24].

### Statistical Analysis

We adopted our VE analysis from the Interim Guidance of Evaluation of COVID-19 Vaccine Effectiveness by the World Health Organization, where the final estimate of absolute VE (aVE) will be determined by calculating the adjusted odds ratio (aOR) for vaccination using the formula aVE = (1 – aOR) × 100% [24]. Unconditional multivariable logistic regression will be used to analyze the aOR of having symptomatic and fatal SARS-CoV-2 infection between the unvaccinated and vaccinated participants. We will perform separate analyses for the Delta- and Omicron-dominant periods. Furthermore, within each period, we will also analyze each vaccinated person separately by their vaccine brand or platform and vaccination doses, such as dose 1, dose 2, or booster. For the main aVE analyses, we will include only vaccinated people with the last dose 1 or dose 2 vaccination date within 14-90 days or with the last booster dose within 7-90 days before their index test date and compare them to the unvaccinated people.

One main logistic model will be assembled by forward inclusion, and only covariates with less than 20% missing data will be considered to be included in the model building. Assessment for confounders and effect modifiers will be performed with the Wald test and by observing the change in the primary exposure coefficient SE. Covariates that act as confounders will be included in the model. If effect modification is present, it is essential to report VE and CIs for each subgroup individually. The linearity between continuous variables and the log odds of having symptomatic SARS-CoV-2 infection will be assessed, and the continuous variables will be modified into categorical variables if necessary. The overall fit of the final multivariate logistic regression model will be assessed with Hosmer and Lemeshow goodness-of-fit test [30], as matching might incur matching-related bias from the cases and controls having similar exposure, thus underestimating the aVE. We will also perform conditional logistic regression as a sensitivity analysis for measuring the aVE estimation against symptomatic SARS-CoV-2 infection or our primary objectives [31,32]. Furthermore, for the analysis of the Omicron period, we will perform an additional analysis of relative VE (rVE) by comparing recipients of one booster dose to recipients of the 2 doses who were eligible to receive the booster dose. The rVE will be calculated using the formula rVE = [(aVE from booster dose – aVE from 2 doses and eligible for booster)/(1 – aVE from 2 doses and eligible for booster)] × 100%. For the rVE analyses, we will include only people whose last booster dose was within 7 to 90 days before their index test date and compare them to people whose last 2-dose vaccination date was ≥90 days before their index test date [24].

This study will also conduct subgroup analyses based on the time between the last vaccination date and test index date to assess for waning of immunity (Table 2). These subgroup analyses will be conducted separately for each wave and vaccine brands or regimens. Similar to the main analysis, each vaccinated subgroup will be compared to unvaccinated people.

**Table 2:** Subgroup analysis based on time since last vaccination.

Subgroup	Description
1	7-14 days between the last vaccination dose and index test date
2	14-28 days between the last vaccination dose and index test date
3	28-56 days between the last vaccination dose and index test date
4	56-90 days between the last vaccination dose and index test date
5	90-180 days between the last vaccination dose and index test date
6	>180 days between the last vaccination dose and index test date

A 2-sided *P* value derived from the multivariable logistic regression analysis will be used to assess VE. Data linkage and data cleaning will be performed in Google Collaboratory with the Python programming language. Furthermore, the Statsmodel library package will be used for statistical analysis [33].

### Ethical Considerations

The research protocol received ethics approval from the Ethical Committee of Persahabatan Hospital Jakarta (40.A.1/KEPK-RSUPP/11/2022). The research conducted in accordance with this protocol will not collect consent from people as it involves the use of secondary data. A consent waiver statement has been issued by the Ethical Committee of Persahabatan Hospital Jakarta (DP.04.03/D.XX.10.4/0001/2024).

## Results

This research received funding from 2022 to 2024. The proposal preparation, protocol development, and ethical review processes began in 2023, alongside efforts to gather access to datasets from various stakeholders. Enrollment commenced at the beginning of 2024 and was completed in July 2024 after the data-cleaning process. A total of approximately 4 million data points were available from the linked datasets. Data analysis is currently underway, with the first results expected to be submitted for publication at the start of 2025.

## Discussion

The general objective of this research is to estimate the real-world effectiveness of COVID-19 vaccines during the Delta and Omicron waves. Since the study covers two different periods, the results may also reveal changes in VE across these two variant phases. The study follows World Health Organization guidelines for conducting real-world COVID-19 vaccine research and incorporates linkage across multiple research datasets to enhance its validity. The use of the Google Collaboratory online platform for data integration demonstrates adaptability to modern technologies. As of mid-2024, there are over 4 million combined records from RT-PCR test results and vaccination data.

Vaccine effectiveness refers to the average response to the vaccine under real-world conditions, assessed through observational studies, and is generally lower than the efficacy observed in controlled settings [34]. When a vaccine is administered to the general population, various factors such as individuals’ medications and overall health status, and vaccine storage and administration conditions, among others, can diminish its effect and increase variability in responses among recipients. This phenomenon is anticipated for all vaccines, underscoring the importance of continuous monitoring for emerging data as vaccination programs progress. The collection, analysis, and communication of these experiences are crucial for gaining authoritative knowledge on effectiveness, including potential rare or delayed side effects. Assessments of COVID-19 VE also contribute to understanding the overall community immunity required to safeguard the population [35].

The study’s inclusion of a wide variety of vaccine platform, including mRNA, adenovirus-based, and inactivated vaccines, is one of its main strengths. For low- and middle-income countries, which may depend on several types of vaccine due to availability constraints, this variety enables a more nuanced knowledge of vaccination performance across various methods. In contrast to many previous studies, this study additionally considers important factors that could affect the chance of vaccination and the risk of contracting or dying from COVID-19 in various communities. The study provides a more complete picture of VE in the real world by taking these factors into account, particularly in different urban environments like Jakarta.

Nevertheless, certain limitations still exist despite the test-negative design study design’s attempts to lessen biases. If those who test negative for COVID-19 were exposed but did not exhibit symptoms, misclassification bias may occur, as indicated in Table 3 on potential biases related to test-negative design. Furthermore, since those who choose to get tested might not be representative of the whole community, health care–seeking behavior may introduce additional bias. Collider bias is still a worry and has not yet been completely mitigated, even though sampling has been used to reduce problems like selection bias. This is especially important because the study looks at communities with different socioeconomic and health characteristics, which could affect the results. Addressing collider bias in future research could significantly improve the reliability and validity of the findings, offering a clearer understanding of the true VE in different contexts.

**Table 3:** Potential biases of COVID-19 vaccine effectiveness studies using test-negative design.

Potential bias	Explanation	Magnitude	Direction on VE^a^ estimate	Outcome/subgroups affected	Method to control	Comments
Health care–seeking behavior bias (access to health care)	Individuals who are more likely to get vaccinated tend to seek medical care more frequently, which increases their chances of being identified as cases	Large	Underestimate VE	Nonsevere outcome	Using TND^b^	By using TND, we only include individuals who seek care for similar symptoms or indications thus reducing differences in health care–seeking behavior, but TND can create collider bias
Health care–seeking behavior bias (vaccine status)	Vaccinated individuals less likely to seek testing for COVID-19–like illness due to perception of protection	Small to moderate	Underestimate VE	Nonsevere outcome	Using TND	TND partially controls this bias
Collider bias	This arises when we restrict analysis on a collider variable (eg, testing). TND only analyzes individuals who were tested.	Unknown	Depends on how health care–seeking behavior and infection affects testing	Nonsevere outcome	Limit to severe individuals or older adults	We could not control this bias because the information on the collider variable (testing) is not available.
Misclassification of the exposure	During high levels of transmissibility, infection may occur soon after vaccination. Meanwhile, vaccines need time to confer an acceptable protective immune response.	High	Underestimate VE	All	Limit analysis only after vaccine performance has acceptable time to confer acceptable protection (eg, 14 days after the first or second dose, 7 days after third dose)	This study excludes infection that occurs <14 days after vaccination in the analysis of primary vaccine (first and second dose) and excludes events (eg, infection) that occur <7 days after vaccination in the analysis of booster vaccines. This measure will prevent underestimation of VE in the time when infection occurs during suboptimal immune response after vaccination.
Misclassification of the outcome	False positive and false negative	Small	Underestimate VE	All	Use only highly sensitive test (eg, RT-PCR^c^)	This study only includes RT-PCR SARS-CoV-2 results (see eligibility criteria) to control this bias
Spurious waning bias	This refers to an apparent VE reduction over time that does not reflect the decay of immunity over time but rather results from biases or confounding factors in the study design or analysis (eg, different variants, differences in exposure risk).	Small to large	Underestimate VE	All	Perform analysis for specific variant/period. Control difference in viral dynamic transmissibility over time. Perform stratification analysis by time since vaccination.	To prevent bias due to variants, this study conducts two sets of analyses (Delta and Omicron). To prevent bias due to differences in exposure risk over the periods of interest, we conducted matching case and control by calendar week of sample collection. To show VE waning, we perform stratification analysis by time since vaccination.

^a^VE: vaccine effectiveness.

^b^TND: test-negative design.

^c^RT-PCR: reverse transcription polymerase chain reaction.

The response to the COVID-19 pandemic has highlighted new challenges in Indonesia’s healthcare system, particularly data fragmentation from numerous health applications and insufficient standardization [36]. Disparities in recording and storing health data in Indonesia also present challenges during the pandemic [37]. Both of these obstacles hinder large-scale health research in the country. To address this, dataset integration is necessary to obtain comprehensive and complete data. Upon completion of our study, it is hoped to demonstrate the capability of health researchers in Indonesia to conduct research using big data, serving as an initiation for the digital transformation of health care.

## Appendix

Multimedia Appendix 1 List source datasets.

## Abbreviations

aOR: adjusted odds ratio
aVE: absolute vaccine effectiveness
NAR: New All Records
PCARE: Primary Care
RT-PCR: reverse transcription polymerase chain reaction
rVE: relative vaccine effectiveness
SUSPROB: Suspect and Probable
VE: vaccine effectiveness

## Data Availability

The data that supports the findings of this study will be available upon request from EB and FA. The data, which contain personal information, are not publicly available due to the strategic health policy of the province of Jakarta.
