# Supplementary material for: Real-World Effectiveness of COVID-19 Vaccines (ChAdOx-1s, CoronaVac, BBIBP-CorV, mRNA-1273, and BNT162b2) in Jakarta: Protocol for Test-Negative Design of Health Care Data
Source: JMIR Res Protoc. 2025 Apr 10;14:e56519. doi: 10.2196/56519 (PMC12022515; doi:10.2196/56519)
Supplement: Multimedia Appendix 1 [file resprot_v14i1e56519_app1.docx]

**Supplementary Files**

Table S1. COVID-19 vaccines distributed in the Special Capital Region of Jakarta during the Delta and Omicron-dominant periods.

| **COVID-19 vaccine brands** | **Vaccination coverage (%) from  the target vaccination aged 18 years and older (N= 8,395,427)** | | |
| --- | --- | --- | --- |
| **During the Delta period (data per 31 August 2021)^1^** | | | |
| **Dose 1 (%)** | | **Dose 2 (%)** | **Booster (%)** |
| SinoVac-CoronaVac Biofarm | 54.45 | 41.29 | 0.01 |
| AstraZeneca  (ChAdOx1 nCoV-19) | 16.60 | 3.79 | 0.00 |
| Covovax-Novovax, (NVX-CoV2373) | 0.00 | 0.00 | 0.00 |
| Johnson&Johnson (Ad26.COV2.S) | 0.00 | 0.00 | 0.00 |
| Moderna  (mRNA-1273) | 0.21 | 0.00 | 0.60 |
| Sinopharm (BBIBP-CorV) | 1.01 | 0.84 | 0.00 |
| Pfizer  (BNT162b2) | 0.49 | 0.01 | 0.00 |
| **During Omicron period (data per 31 March 2022)^1^** | | | |
| SinoVac-CoronaVac Biofarm | 76.73 | 69.62 | 0.00 |
| AstraZeneca  (ChAdOx1 nCoV-19) | 18.40 | 16.03 | 9.36 |
| Covovax-Novovax, Indovax (NVX-CoV2373) | 0.04 | 0.04 | 0.02 |
| Johnson&Johnson (Ad26.COV2.S) | 0.02 | 0.00 | 0.00 |
| Moderna  (mRNA-1273) | 1.01 | 0.84 | 2.69 |
| Sinopharm (BBIBP-CorV) | 6.89 | 5.78 | 11.35 |

Table S2. Example of final dataset after linkage and integration (separate dataset for each of the Delta and Omicron periods)

| **Name of variables** | **Details** |
| --- | --- |
| ID Number | Will be de-identified |
| Male | Yes = 1, otherwise 0 |
| Date of birth | dd/mm/yyyy (datetime format) |
| Date of index test | dd/mm/yyyy (datetime format) |
| Week of PCR collection | Whole number starting at 1.  For the Delta 1–7 August 2021 served as week 1, while 1 January 2022 served as week 1 in the Omicron period. |
| Period of interest | Omicron or Delta |
| Age in years at diagnosis | Age in years |
| Case or positive result | Yes = 1, otherwise 0 |
| Occupation | Civilians = 0, public/goverment officials=1, healthcare workers = 2 |
| Domicile at district level | Categorical variables: West Jakarta, East Jakarta, North Jakarta, South Jakarta, Central Jakarta, dan Kepulauan Seribu |
| Living in impoverished neighborhood | Yes = 1, otherwise 0 |
| Presence of any comorbidities | Yes = 1, otherwise 0 |
| Death case | Yes = 1, otherwise 0 |
| Vaccinated | Yes = 1, otherwise 0 |
| Date of first vaccination | dd/mm/yyyy (datetime format)  N/A if unvaccinated |
| Name of the first vaccine | Categorical variable |
| Date of second vaccination | dd/mm/yyyy (datetime format)  N/A if unvaccinated |
| Name of the second vaccine | Categorical variable |
| Date of booster vaccination | dd/mm/yyyy (datetime format)  N/A if unvaccinated |
| Name of the booster vaccine | Categorical variable |
| Last vaccination dose | Categorical variable (unvaccinated, dose 1, dose 2, and booster) |
| Time since last vaccination | Days between last vaccination dose to date of test date |
| Previous COVID-19 infection | Yes = 1, otherwise 0 |

Table S3. Example of the matched test-negative case control dataset

| **Name of variables** | **Details** |
| --- | --- |
| ID Number | Will be de-identified |
| Male | Yes = 1, otherwise 0 |
| Week of PCR collection | Whole number starting at 1.  For the Delta 1–7 August 2021 served as week 1, while 1 January 2022 served as week 1 in the Omicron period. |
| Period of interest | Omicron or Delta |
| Age in years at diagnosis | Age in years |
| Case or positive result | Yes = 1, otherwise 0 |
| Set ID | Matched controls will be represented by the case ID number |
| match_counts | Number of controls within each matched sets (can be 0, 1, or 2) |
| Vaccinated | Yes = 1, otherwise 0 |
| Last vaccination dose | Categorical variable (unvaccinated, dose 1, dose 2, and booster) |
| Name of the last vaccine brands | Categorical variable |
| Time since last vaccination | Days between last vaccination dose to date of test date |
| Previous COVID-19 infection | Yes = 1, otherwise 0 |
| Occupation | Civilians = 0, public/goverment officials=1, healthcare workers = 2 |
| Domicile at district level | Categorical variables: West Jakarta, East Jakarta, North Jakarta, South Jakarta, Central Jakarta, dan Kepulauan Seribu |
| Living in impoverished neighborhood | Yes = 1, otherwise 0 |
| Presence of any comorbidities | Yes = 1, otherwise 0 |

Table S4. Minimum number of cases and controls to detect a specified vaccine effectiveness, for an estimated vaccination coverage in the population under evaluation, with 2 controls per cases, with the precision of +5%, and a type 1 error rate of 0.50 based on the methodology by O’Neill^2,3^

| Anticipated vaccine effectiveness | Vaccination coverage in studied population | Minimum no of cases | Minimum no of controls |
| --- | --- | --- | --- |
| 50% | 20% | 5,107 | 10,214 |
|  | 30% | 3,570 | 7,140 |
|  | 40% | 2,859 | 5,718 |
|  | 50% | 2,506 | 5,012 |
|  | 60% | 2,377 | 2,754 |
|  | 70% | 2,469 | 4,938 |
|  | 80% | 2,939 | 5,878 |
|  | 90% | 4,733 | 9,466 |
| 60% | 20% | 3,763 | 7,526 |
|  | 30% | 2,567 | 5,134 |
|  | 40% | 2,002 | 4,004 |
|  | 50% | 1,706 | 3,412 |
|  | 60% | 1,570 | 3,140 |
|  | 70% | 1,579 | 3,158 |
|  | 80% | 1,817 | 3,634 |
|  | 90% | 2,826 | 5,652 |
| 70% | 20% | 2,587 | 5,174 |
|  | 30% | 1,715 | 3,430 |
|  | 40% | 1,296 | 2,592 |
|  | 50% | 1,066 | 2,132 |
|  | 60% | 943 | 1,886 |
|  | 70% | 909 | 1,818 |
|  | 80% | 1,000 | 2,000 |
|  | 90% | 1,484 | 2,968 |
| 80% | 20% | 1,580 | 3,160 |
|  | 30% | 1,013 | 2,026 |
|  | 40% | 735 | 1,470 |
|  | 50% | 578 | 1,156 |
|  | 60% | 485 | 970 |
|  | 70% | 439 | 878 |
|  | 80% | 451 | 902 |
|  | 90% | 622 | 1,244 |
| 90% | 20% | 749 | 1,498 |
|  | 30% | 461 | 922 |
|  | 40% | 318 | 636 |
|  | 50% | 234 | 468 |
|  | 60% | 181 | 362 |
|  | 70% | 148 | 296 |
|  | 80% | 134 | 268 |
|  | 90% | 159 | 318 |

Reference:

1. Ministry of Health, Republic Indonesia. COVID-19 vaccination report [In Bahasa]. Available from:https://layanandata.kemkes.go.id/katalog-data/covid-19/visualisasi-data/laporan-vaksinasi-covid-19. Accessed October 2024
2. O’Neill RT. On sample sizes to estimate the protective efficacy of a vaccine. Stat Med. 1988;7:1279–88
3. World health organization. Evaluation of COVID-19 vaccine effectiveness, interim guidance. 2021
